# Next-Generation Sequencing and the CRISPR-Cas Nexus: A Molecular Plant Virology Perspective

**DOI:** 10.3389/fmicb.2020.609376

**Published:** 2021-01-12

**Authors:** Muhammad Shafiq Shahid, Muhammad Naeem Sattar, Zafar Iqbal, Amir Raza, Abdullah M. Al-Sadi

**Affiliations:** ^1^Department of Plant Sciences, College of Agricultural and Marine Sciences, Sultan Qaboos University, Muscat, Oman; ^2^Central Laboratories, King Faisal University, Hofuf, Saudi Arabia

**Keywords:** CRISPR, CRISPR associated (Cas) proteins, genome editing, next generation sequencing (NGS), plant viruses

## Abstract

In recent years, next-generation sequencing (NGS) and contemporary Clustered Regularly Interspaced Short Palindromic Repeats (CRISPR)-CRISPR-associated (Cas) technologies have revolutionized the life sciences and the field of plant virology. Both these technologies offer an unparalleled platform for sequencing and deciphering viral metagenomes promptly. Over the past two decades, NGS technologies have improved enormously and have impacted plant virology. NGS has enabled the detection of plant viruses that were previously undetectable by conventional approaches, such as quarantine and archeological plant samples, and has helped to track the evolutionary footprints of viral pathogens. The CRISPR-Cas-based genome editing (GE) and detection techniques have enabled the development of effective approaches to virus resistance. Different versions of CRISPR-Cas have been employed to successfully confer resistance against diverse plant viruses by directly targeting the virus genome or indirectly editing certain host susceptibility factors. Applications of CRISPR-Cas systems include targeted insertion and/or deletion, site-directed mutagenesis, induction/expression/repression of the gene(s), epigenome re-modeling, and SNPs detection. The CRISPR-Cas toolbox has been equipped with precision GE tools to engineer the target genome with and without double-stranded (ds) breaks or donor templates. This technique has also enabled the generation of transgene-free genetically engineered plants, DNA repair, base substitution, prime editing, detection of small molecules, and biosensing in plant virology. This review discusses the utilities, advantages, applications, bottlenecks of NGS, and CRISPR-Cas in plant virology.

## Introduction

Plant viruses infect diverse plant species across the globe. During successful infections, viruses trigger an array of interactions with insect vectors or the plant hosts. Some plant viruses have acquired extra-viral components, *viz*., DNA-satellites, RNA-satellites, and satellite viruses (Mansoor et al., 1999; Briddon et al., 2001; Palukaitis, 2016). Plant viruses have caused a significant reduction in crop productivity across Asia, Africa, Europe, and South America, resulting in losses of approximately 30 billion US$ annually (Sastry and Zitter, 2014). For example, in the last decade, cassava mosaic disease caused an approximate 25 million ton reduction in cassava production worldwide (Legg and Thresh, 2000; Thresh and Cooter, 2005). Millions of citrus plants have been destroyed annually by the Citrus Tristeza virus (CTV) (Moreno et al., 2008; Harper, 2013). The potato leafroll virus has led to a loss of approximately US$100 million in the United States and around £50 million in the United Kingdom (Wale et al., 2008; Sastry and Zitter, 2014). Similarly, during the period between 1992 and 9197, cotton leaf curl disease caused a loss of around US$5 billion (Briddon et al., 2001) to Pakistan’s economy.

An effective approach to virus control requires efficient detection methods and subsequent insights into the genomic architecture of the target viruses. Various approaches, including enzyme-linked immunosorbent assay (ELISA), restriction enzyme analysis, polymerase chain reaction (PCR), and reverse transcriptase PCR (RT-PCR), have been regularly used as an initial screening tool. Most of these diagnostic techniques rely on prior knowledge of viral genomes so that unknown viruses may remain undetected. Next-generation sequencing (NGS) technologies have revolutionized the field of molecular biology, especially plant virology, by comprehensively unearthing the genomic data at a level that was not possible before. Contemporary NGS technologies can sequence all types of nucleic acid molecules, concurrently. NGS technologies have enabled the detection of novel pathogenic viruses that have remained undetected due to low viral titer or detection threshold levels (Villamor et al., 2019). These NGS technologies facilitate the discovery of overlooked plant virus species and help broaden our understanding of phytoviromes.

Recombination has been used as a tool to modify prokaryotic genomes, but this approach was least specific and low yielding. The discovery of four sequence-specific endonucleases such as meganucleases, Zinc Finger Nuclease (ZFN), Transcription Activator like Effector Nuclease (TALEN), and Clustered Regularly Interspaced Short Palindromic Repeats (CRISPR)-CRISPR-associated protein 9 (CRISPR-Cas9) (Zhang et al., 2013) substantially improved the genome editing (GE) in higher organisms (Wiedenheft et al., 2011; Jinek et al., 2012, 2013; Gaj et al., 2013). Among them, the CRISPR-Cas system is the simplest, most efficient, and versatile GE tool that allows site-directed mutagenesis at the desired genomic position (Gaj et al., 2013; Bortesi and Fischer, 2015).

**FIGURE 1:**
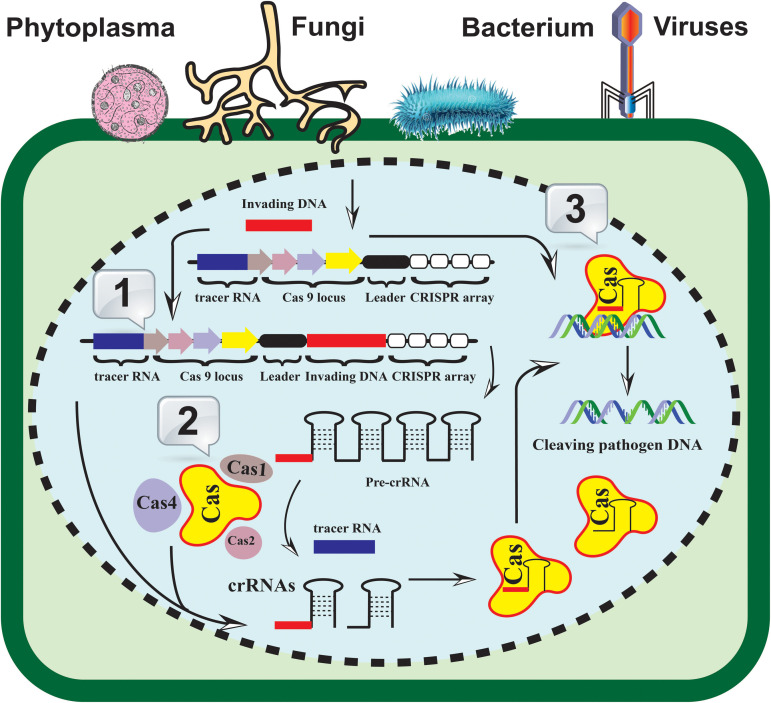
A generalized CRISPR-Cas-based resistance induction model. The identification, recruitment, and cleavage of the invading pathogen’s nucleic acid (fungi, bacteria, viruses, and/or phytoplasma) are achieved in three fundamental steps. 1. Acquisition: the invaded DNA (bar in red color) is integrated into the CRISPR-array (white rectangles) as new spacers; 2. Expression: the pre-CRISPR and crRNAs machinery is triggered and expressed in the invaded cell; 3. Interference: the mature crRNA hybridized to the invading pathogen genome and subsequently recognized by the Cas proteins. The coordination of Cas helicase and nuclease with CRISPR RNA machinery results in the invader’s nucleic acid cleavage.

CRISPR-Cas systems are derived from prokaryotic immune systems that provide indigenous immunity against invading nucleic acids (Figure 1). CRISPR-Cas systems are diverse and can be divided into two major classes, six different types, and multiple types (Makarova et al., 2020). The main components of the most widely used CRISPR-Cas systems are Cas9 endonucleases, which are derived from different microorganisms, such as *Streptococcus pyogenes*, *Staphylococcus aureus*, and *Francisella novicida*, and are part of class II type II systems (Makarova and Koonin, 2015; Makarova et al., 2020). These Cas9 proteins are accompanied by CRISPR-RNA (crRNA) and trans-activating CRISPR-RNA (tracrRNA) for sequence-dependent cleavage of foreign nucleic acids (Hille et al., 2018; Makarova et al., 2020). The invention of a single-guide RNA (sgRNA) increased the potential applications of CRISPR-Cas systems (Jinek et al., 2012). As an initial step in CRISPR-Cas-based prokaryotic immunity, nucleic acid fragments of the invading pathogens are integrated into the CRISPR locus during infection. The subsequent infections thus activate the transcription of these smaller fragments as part of the CRISPR array they coordinate with CRISPR-associated (Cas) protein machinery to recognize, bind, and cleave to the foreign DNA/RNA elements.

In addition to the well-known CRISPR-Cas9 systems, the biotech applications for a number of the other CRISPR types and subtypes are also under active development. CRISPR-Cas systems are classified into two major classes based on interference type: Class I and Class II. Class I and II are further categorized into six types based on the type of nucleic acid they target (Makarova and Koonin, 2015). The evolutionary classification of CRISPR-Cas systems, especially class II and its variants, has been appraised by Makarova et al. (2020). Among the class II single Cas protein systems, these include Cas12 (type V), Cas9 (type II), Cas13a-d (type VI), and Cas14a-c (type V-F) (Burstein et al., 2017; Shmakov et al., 2017). Like Cas9, Cas12 targets double-stranded (ds) DNA, however, Cas13 (type VI) effector proteins: Cas13a (C2c2) (Abudayyeh et al., 2016), Cas13b (C2c6) (Cox et al., 2017), Cas13c (C2c7) (Shmakov et al., 2017), and Cas13d (Yan et al., 2018) are characterized as RNA-guided ribonucleases in microbial genomes.

In contrast to the class 2 systems, class 1 systems are composed of multiple Cas proteins, in different combinations, depending on the type and sub-type. Specifically, type I complexes are generally composed of Cas5, Cas6, Cas7, Cas8, and Cas11, and target dsDNA, while the type III complexes are composed of Cas5, Cas6, Cas8, and Cas10 and these are widespread in the immune system of nearly a quarter of bacterial species (Koonin et al., 2017). Type III CRISPR is further divided into two subtypes, i.e., type III-A and type III-B, based on two main Cas effectors (Cas10-Csm and Cas10-Cmr). Type II are composed of Cas5, Cas6, Cas7, and Csf1. Interestingly, these type III systems do not require a PAM, and instead, target nascent mRNAs and the corresponding DNA in transcriptionally active complexes.

In the last few years, the applications of CRISPR-Cas technology have been extended to all the fields of bioscience, including animal and human cell lines (Belhaj et al., 2015), as well as human viruses (both RNA and DNA) (Hadidi et al., 2016). CRISPR technology has extensive applications encompassing the insertion and/or deletion of a particular segment of DNA, introducing site-directed mutagenesis, expression and/or repression of genes, and epigenome remodeling. CRISPR-Cas systems offer great advantages including ease of cloning, low cost, and multiplexing, where multiple sites in the genome can be targeted simultaneously (Shin et al., 2017; Manghwar et al., 2019; Lee et al., 2020). Several studies have demonstrated the effectiveness of CRISPR-Cas technology due to its fast, easy to use applications in recalcitrant species, which can be effectively used to introduce or remove different genes (at a time) and do not require many manipulating tools. Since the first application of CRISPR-Cas-based GE in plants in 2013, this technique has been used continuously to engineer resistance against a variety of plant viruses (Scheben et al., 2017; Vats et al., 2019; El-Mounadi et al., 2020).

Three general approaches to engineering anti-viral mechanisms using CRISPR-Cas have been suggested: (1) the Cas9/sgRNA complex recruits viral genetic elements such as the origin of replication (ori) and averts the binding of replication-associated proteins, (2) the Cas9/sgRNA complex directly dissects the viral nucleic acid to prevent viral DNA replication, and (3) the Cas9/sgRNA complex mutates the viral genome at certain positions through non-homologous end joining (NHEJ) (Chaparro-Garcia et al., 2015). In addition to Cas9, a recently discovered endonuclease Cas12a (formerly referred to as Cpf1) offers dual nuclease activity as an endoribonuclease for crRNA processing and as endo-deoxyribonuclease to dissect the nucleic acid and produce ds breaks (DSB), respectively (Alok et al., 2020). Another milestone in CRISPR-Cas-based GE is the recent discovery of another effector protein, Cas13, an RNAi analog in eukaryotes for directly targeting the RNA genomes of plant viruses (Abudayyeh et al., 2017). This system has not been exploited yet against DNA viruses. However, it can be used to target the mRNA of DNA viruses during infection (Loriato et al., 2020). In geminiviruses, the CRISPR-Cas system can be employed to effectively inhibit virus accumulation by targeting any genomic region and stacking different sgRNAs against a single virus or multiple viruses and their associated DNA-satellites (Iqbal et al., 2016; Rahman et al., 2017; Dahan−Meir et al., 2018; Ali et al., 2019; Roy et al., 2019).

Advances in NGS and GE technologies are revolutionizing the fields of genetics, genomics, molecular biology, and others, including plant virology. NGS technologies helped in the emergence and evolution of modern gene silencing and GE technologies, such as RNA interference (RNAi) and CRISPR-Cas, respectively. In this review article, we discuss the role of NGS and CRISPR-Cas technologies in plant virology.

## Next-Generation Sequencing Technologies

A huge effort was made to sequence the entire human genome, which cost about US$ 3 billion and took nearly a decade to complete using the Sanger sequencing platform. NGS technologies have empowered the processing of large genomic datasets that generate an enormous amount of sequence reads and reduce costs to less than US$ 1000 per genome. Other advantages of NGS include the quantification of gene expression, the discovery of new RNA species such as microRNA (miRNA) regulating gene expression, metagenomic analysis, identification of latent viruses, multiplexing, and the ability to decipher specific species or strain of virus(es) without prior knowledge (Mardis, 2011). The development of new software with improved bioinformatics algorithms has also contributed to revolutionizing NGS techniques. NGS techniques have been categorized from first-to-fourth generations and are briefly discussed in the subsequent sections.

## Evolution of NGS Technologies and Their Role in Virus Discovery

Part of the first generation of sequencing technologies, Sanger and Maxam-Gilbert’s invention of the first DNA sequencers revolutionized the field of molecular biology (Sanger et al., 1977).

### First-Generation Sequencing Platforms

The first automated Sanger sequencing platform with single capillary electrophoresis (ABI Prism 310) was made commercially available in the mid-1990s (Watts and MacBeath, 2001). It was subsequently upgraded to a pre-NGS DNA technology with 96 lanes capillary electrophoresis (Paegel et al., 2002) to fix the issues associated with cost, time, and quality of the sequencing output (Metzker, 2005). For the last four decades, the Sanger sequencing technique is the preferred method for researchers dealing with the smaller DNA genomes of plant viruses. The ABI 3730xl DNA sequencer can generate reads from a genome as small as 1.9 kb to as long as 84 kb with a maximum precision of 99.99% and an average read length of 400–900 bp in about 3 h run-time (Liu et al., 2012). The impediments related to the Sanger sequencing platforms, including low scalability, tediousness, and cost ineffectiveness, have been developed in present-day cutting-edge sequencing technologies.

### Second-Generation Sequencing Platforms

To address the shortcomings of the Sanger sequencing technique, second-generation sequencing technologies were released in 2005. They were later owned and commercially launched by Roche in 2007. These sequencing platforms not only reduced sequencing costs but produced millions of short reads (Kchouk et al., 2017); the Roche 454 platform worked on the principle of emulsion polymerase-mediated nucleotide extension and utilized sequencing-by-synthesis chemistry, which captures a template molecule, loads it into the well, and sequences the template genome on the basis of parallel pyrosequencing (Rothberg et al., 2011). The latest 454 FLX Titanium XL + framework can deliver around one million reads in a single run spanning about 23 h and having read lengths of about 700 bp with a maximum accuracy of 99.997% (Table 1).

**TABLE 1:** Next-generation sequencing (NGS)-mediated discovery of selected different plant infecting viruses.

Sequencing platform	Virus identified*	Genome	Virus taxonomy	Host plant	Region	References
Illumina	CLCuGV, ToLCSDV, TYLCV-OM	ssDNA	Begomovirus (*Geminiviridae*)	Tomato and okra	Saudi Arabia	Idris et al., 2014
	SqMV	(+)ssRNA	Comovirus (*Secoviridae*)	Squash	Spain	Li et al., 2015
	SbBMV, ToYSV, ToYVSV, ToDfLV, SiGMBRV, PepBLV	ssDNA	Begomovirus (*Geminiviridae*)	Pepper	Argentina	Bornancini et al., 2020
	ToMV	(+)ssRNA	Tobamovirus (*Virgaviridae*)	Chickpea	Netherlands	Pirovano et al., 2015
	AGV	ssDNA	*Geminiviridae*	Apple	China	Liang et al., 2015
	MMDaV, CCDaV	ssDNA	Mastrevirus and Becurtovirus (*Geminiviridae*)	Mulberry	China	Ma et al., 2015
	CuLCrV	ssDNA	Begomovirus (*Geminiviridae*)	*B. tabaci*	Florida	Ng et al., 2011
	TPNRBV, TPLPV	(+)ssRNA	Blunervirus and Ilarvirus (*Bromoviridae*)	Tea	China	Hao et al., 2018
	NSPaV	(+)ssRNA	Luteovirus (*Luteoviridae*)	Nectarine	California	Bag et al., 2015
	CYLV	(+)ssRNA	Closterovirus (*Closteroviridae*)	Carrot	United Kingdom	Adams et al., 2014
	CCDaV	ssDNA	*Geminiviridae*	Citrus	Italy	Loconsole et al., 2012b
	PPSMV	(-)ssRNA	Emaravirus (*Luteoviridae*)	Pigeon pea	Italy	Elbeaino et al., 2014
	GRLaV	ssDNA	Grablovirus (*Geminiviridae*)	Grape	California	Poojari et al., 2013
	CVEV	(-)ssRNA	Emaravirus (*Luteoviridae*)	Citrus	Spain	Vives et al., 2013
	MYMIV	ssDNA	Begomovirus (*Geminiviridae*)	Tomato	Oman	Shahid et al., 2019
	SsHADV-1	ssDNA	Gemycircularvirus (*Genomoviridae*)	Dragonfly	Arizona/Oklahoma	Dayaram et al., 2015
	LCV, TCV, CpMMV	(+)ssRNA	Criniviruses (*Closteroviridae*) and Carlavirus (*Betaflexiviridae*)	*B. tabaci*	Florida	Rosario et al., 2014
Roche 454	BVF	dsDNA	Badnavirus (*Caulimoviridae*)	Blackberry	Arkansas	Shahid et al., 2017
	LChV-1	(+)ssRNA	Velarivirus (*Closteroviridae*)	Cherry	France	Candresse et al., 2013
	GMMV	(+)ssRNA	Cucumovirus (*Bromoviridae*)	Tomato	United Kingdom	Adams et al., 2009
	RLBV	(-)ssRNA	Emaravirus (*Luteoviridae*)	raspberry	United Kingdom	McGavin et al., 2012
	SWSV	ssDNA	Mastrevirus (*Geminiviridae*)	Sugarcane	Egypt	Candresse et al., 2014
	RRV	(-)ssRNA	Emaravirus (*Luteoviridae*)	Rose	Arkansas	Laney et al., 2011
Oxford Nanopore [MinION]	PVS, PVX, PVY, PLRV	(+)ssRNA	Carlavirus (*Betaflexiviridae*), Potexvirus (*Alphaflexiviridae*), Potyvirus (*Potyviridae*), Polerovirus (*Luteoviridae*)	Potato	Ireland	Della Bartola et al., 2020
	CMV	ssDNA	Begomovirus (*Geminiviridae*)	Cassava	Tanzania, Uganda, and Kenya	Boykin et al., 2019
	WSMV, TriMV, BYDV	(+)ssRNA	Poacevirus and Tritimovirus (*Potyviridae*), Luteovirus (*Luteoviridae*)	Wheat	United States	Fellers et al., 2019
	PPV	(+)ssRNA	Potyvirus (*Potyviridae*)	Plant tissue	United States	Bronzato Badial et al., 2018

*Selected viruses are included in each case and acronyms used are: apple geminivirus (AGV), barley yellow dwarf virus (BYDV), blackberry virus F (BVF), carrot yellow leaf virus (CYLV), cassava mosaic viruses (CMV), citrus vein enation virus (CVEV), citrus yellow vein clearing virus (CYVCV), citrus chlorotic dwarf-associated (CCDaV), cucurbit leaf crumple virus (CuLCrV), cotton leaf curl Gezira virus (CLCuGV), cowpea mild mottle virus (CpMMV), gayfeather mild mosaic virus (GMMV), grapevine red leaf-associated virus (GRLaV), lettuce chlorosis virus (LCV), little cherry virus 1(LChV-1), mulberry mosaic dwarf-associated virus (MMDaV), mungbean yellow mosaic India virus (MYMIV), nectarine stem pitting-associated virus (NSPaV), pepper blistering leaf virus (PepBLV), pigeon pea sterility mosaic virus (PPSMV), plum pox virus (PPV), potato virus Y (PVY), potato virus X (PVX), potato virus S (PVS), potato leafroll virus (LRV), raspberry leaf blotch virus (RLBV), riticum mosaic virus (TriMV), rose rosette virus (RRV), sclerotinia sclerotiorum hypovirulence-associated DNA virus-1 (SsHADV-1), Sida golden mosaic Brazil virus (SiGMBRV), Soybean blistering mosaic virus (SbBMV), squash mosaic virus (SqMV), sugarcane white streak virus (SWSV), tea plant necrotic ring blotch virus (TPNRBV), tea plant line pattern virus (TPLPV), tomato leaf curl Sudan virus (ToLCSDV), tomato yellow leaf cur virus-Oman (TYLCV-OM), tomato yellow spot virus (ToYSV), tomato yellow vein streak virus (ToYVSV), tomato dwarf leaf virus (ToDfLV), tomato mottle mosaic virus (ToMV), tomato chlorosis virus (TCV), and wheat streak mosaic virus (WSMV).

The Solexa Genome Analyzer, introduced in 2006 and later acquired by Illumina in 2007, was another addition to the second-generation sequencing platform (Balasubramanian, 2015). Improvements in high-throughput sequencing, a less than 1% error rate, and substantial reductions in sequencing costs made Illumina sequencers the most suitable and frequently used NGS platforms (Fox et al., 2014; Pfeiffer et al., 2018). Apple stem grooving virus, blackberry chlorotic ringspot virus, and prunus necrotic ringspot virus, in addition to a previously unknown rose-leaf rosette-associated virus, were identified as infecting rose plants using the Illumina-Solexa sequencing platform (He et al., 2015). Recently, a mixed infection of tomato yellow leaf cur virus (TYLCV), mungbean yellow mosaic virus (MYMIV), and an associated tomato leaf curl betasatellite was identified in tomato plants using the Illumina HighSeq 4000PE101 platform (Shahid et al., 2019). Liang et al. (2015) employed the Illumina HiSeq 2000 sequencing platform to detect a novel apple geminivirus (AGV) infecting apple trees in China. Sequencing by Oligo Ligation Detection (SOLiD) is another second-generation platform with a throughput of around 9 gigabytes (GB) per run and an average read length of 50 bp. The output of the available SOLiD platforms can also be increased using a quadrant and/or octet slide platform through multiplexing. The high-end SOLiD 5500xl system offers 180 GB output data per run and an improved read length of 2 × 60 bp and produces ca. 3 billion paired-end reads in 24 h with 99.99% accuracy. Another second-generation sequencing platform, the Ion Torrent Semiconductor system, is based on the principle of detecting the released H^+^ ions and is the most suitable platform for microbiome and exome sequencing (Rothberg et al., 2011).

### Third-Generation Sequencing Platforms

The development of third-generation sequencing technologies has led to many problems associated with second-generation sequencing systems, including intensive sample preparation, PCR-based amplification, and sequencing time. These technologies use the single molecule real time (SMRT) sequencing method, which uses fluorescently labeled nucleotide bases that fluoresce upon incorporation into the growing DNA read. The PacBio sequencing system is a third-generation SMRT sequencing system, which requires less than 5 h for sample preparation with a much-reduced cost, and produces an average read length of around 10 kb (Liu et al., 2012; Chin et al., 2016; Kchouk et al., 2017). The only drawback is the high error rate (14%) in the PacBio system, which has been resolved by recurrent sequencing of a single DNA molecule using hairpin adaptors to generate a circular ds DNA template and producing 30x consensus sequences with a superior accuracy of about 99.99%. Oxford Nanopore (*viz*., MinION and PromethION) are single-molecule sequencing technologies developed by Oxford Nanopore Technologies (ONT). This technology provides not only better resolution but also long reads of superior quality and MinION is also suitable for single-nucleotide polymorphisms (Greig et al., 2019). The PromethION system can give an ∼8.5 TB and produces high-quality long reads of around 10 kb at a very low cost. Although the error rate is very high (∼10–15%), it is still being implemented in various research areas (Rang et al., 2018). ONT was successfully exploited for the identification of begomoviruses causing cassava mosaic disease in cassava plants in sub-Saharan African countries (Boykin et al., 2019) (Table 1).

### Fourth-Generation Sequencing Platforms

Subsequent advances in NGS technologies resolved the shortcomings of preceding platforms. The recently developed, *in situ* sequencing (ISS) is a fourth-generation sequencing platform that directly sequences the nucleic acids by spatially resolving the transcriptomics of cells and tissues (Mignardi and Nilsson, 2014). ISS offers a great advantage by revealing distinctive variations, even at the single nucleotide level (Ke et al., 2016). Despite the limitations associated with each platform, there is an appropriate platform for each experimental requirement. Fourth-generation sequencing platforms have not yet been employed for plant virus detection but show potential and it is anticipated that future studies will continue to develop the potentials of this technology in this area.

### Application of NGS to Deciphering the Role of miRNAs in Plant–Virus Interactions

Non-coding small RNAs (sRNAs), including miRNAs and short interfering RNAs (siRNAs), comprise 20–30 nt long molecules that play regulatory roles in plant–virus interactions. For instance, the expressions of miR156, miR158, miR160, miR164, and miR1885 were elicited during turnip mosaic virus (TuMV) infection (He et al., 2008) and miR162 was upregulated during cotton leafroll dwarf polerovirus (CLRDV) infection (Silva et al., 2011). The upregulation of miR444 and downregulation of miR528 and miR396 in rice, wheat, barley, sorghum, and sugarcane during rice stripe virus (RSV) infection are some striking examples (Zhang et al., 2019). A common miRNA, miR168, is upregulated by tobacco mosaic virus (TMV), potato virus X (PVX), and tobacco etch virus (TEV) infection (Várallyay et al., 2010). Exploration of virus-responsive sRNA profiles is crucial for unraveling plant–virus interactions.

Many sRNA molecules, including transacting siRNA, phased siRNA, and the repeat-associated siRNA, are substantial contributors in mediating plant host–virus interactions. NGS revealed the changes in the miRNAs expression profile of miR156, miR159, miR160, miR166, miR398, miR1511, miR1514, and miR2118 upon MYMIV infection in *Vigna mungo* plants, while four novel miRNAs, *viz*., vmu-miRn7, vmu-miRn8, vmu-miRn13, and vmu-miRn14, were also identified (Kundu et al., 2017). The NGS-based analysis of the miRNA profiles of two tomato varieties, Pusa Ruby and LA1777, upon tomato leaf curl virus infection, led to the identification of 53 novel miRNAs, 15 novel homologs, and 91 already known miRNAs (Tripathi et al., 2018). Another class of RNAs, long non-coding RNAs (lncRNAs), also play a pivotal role in host–virus interactions and can originate from either virus, plant, or both (Fortes and Morris, 2016). The functions of many sRNA species have not been explored yet but may contribute to better plant protection strategies. A complete virome analysis is a prerequisite to fully explore the sRNA profile of a plant with a mixed virus infection. Without the assistance of modern NGS technology, the analysis of complete virome would be a time-consuming, tedious, and chaotic task.

Grapevine samples collected from a vineyard in South Africa were analyzed by deep sequencing of total RNAs using the Illumina Genome Analyzer platform. This process successfully detected grapevine leafroll-associated virus 3 (GLRaV-3), grapevine rupestris stem pitting associated virus, and grapevine virus A (Coetzee et al., 2010). In another study, the *de novo* genome assembly of virus enriched sRNAs using Illumina Genome Analyzer IIx platform led to the discovery of a novel potyvirus tomato necrotic stunt virus (ToNSV) from tomato (Li et al., 2012). A complete virome of pepper (*Capsicum* species) plants comprising aphid lethal paralysis virus, bell pepper endornavirus, chilli leaf curl virus (ChiLCV), pea streak virus, pepper leaf curl Bangladesh virus, tobacco vein clearing virus, and a novel pepper virus A was reported using Illumina’s HiSeq 2000 platform (Ng et al., 2011; Candresse et al., 2014; Dayaram et al., 2015; Liang et al., 2015; Jo et al., 2017; Hao et al., 2018; Bornancini et al., 2020). The development of sequencing platforms together with the application of bioinformatics tools proved to be a robust approach for the precise detection of viruses (Fonseca et al., 2018).

A metagenomics study unraveled the presence of sida golden mosaic Brazil virus, soybean blistering mosaic virus, tomato dwarf leaf virus, tomato yellow spot virus, tomato yellow vein streak virus, and a novel pepper blistering leaf virus in a single infection of pepper plants in Argentina (Bornancini et al., 2020). A vine plant exhibiting grapevine viral disease symptoms was subjected to NGS analysis, revealing a mixed infection involving different RNA viruses (Al Rwahnih et al., 2009). The study assessed the genomic and biological properties of a potyvirus, Bean yellow mosaic virus, isolated from *Lupinus angustifolius* plants with mild to severe symptoms, and two other plant species using the Illumina HiSeq2000 platform. The results showed the presence of one new virus and 23 new BYMV sequences. Based on these newly identified sequences, the phylogenetic evolutionary relationship was inferred, and a new nomenclature was proposed (Kehoe et al., 2014). Over the last few years, hundreds of new plant virus species have been reported through NGS technology (Barba et al., 2014; Ho and Tzanetakis, 2014; Roossinck et al., 2015; Wu et al., 2015). NGS has also been extremely useful in determining the host range and genetic diversity of plant viruses and in understanding their evolution (Roossinck, 2017). NGS has been employed to determine the mutational landscapes, particularly SNPs in the genomes of plant viruses (Kutnjak et al., 2017; Katsiani et al., 2020). Studying the genetic diversity of plant viruses helps devise robust strategies to circumvent viral infections and differentiate prevalent viral strains and discover new viral isolates (Marais et al., 2014).

## NGS: Impact on Quarantine Plant and Virus Characterization

The discovery and detection of new/unknown viruses have been augmented with the advent of NGS technologies. Prior to the execution of NGS, pistachio rosette virus was the only virus known to infect pistachio trees in Russia and Iran (Kreutzberg, 1940), but the NGS analysis revealed the presence of a new virus together with a virus-like agent provisionally named “pistachio ampelovirus A” and citrus bark cracking viroid-pistachio, respectively (Al Rwahnih et al., 2018).

**FIGURE 2:**
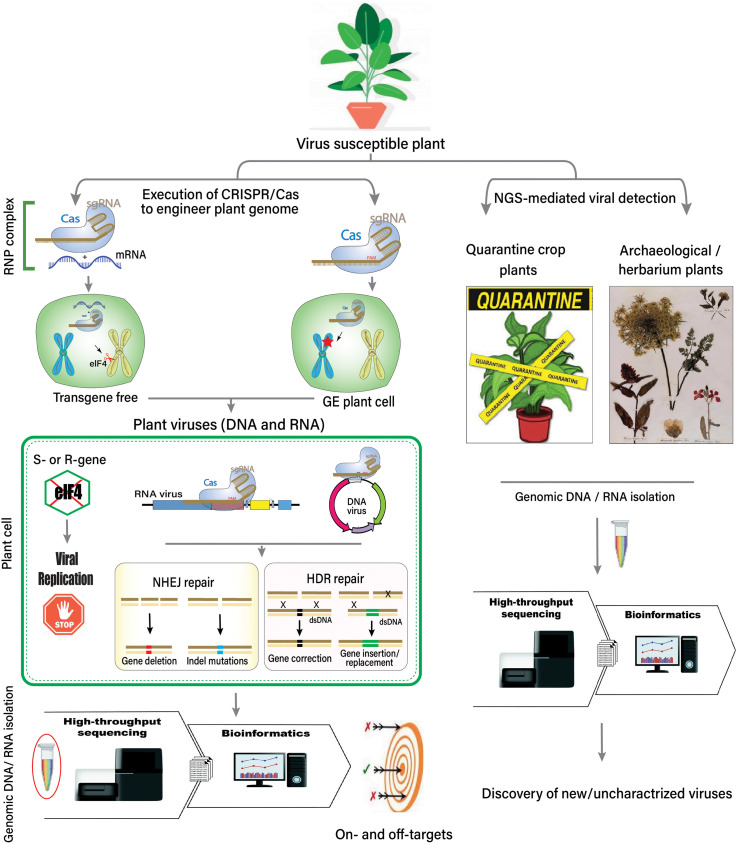
Overview of the host–virus interaction, the role of HTS, CRISPR-Cas system, and the discovery of unknown viruses.

It has been predicted that NGS will be used to monitor or control the dissemination of plant viruses and/or infected plant material across borders through quarantine measures (Figure 2). The increasing volume of trade, exchange of germplasm, and the diversity of plant material are significant threats due to the movement of plant viruses and phytopathogens across the globe. Plant quarantine and certification programs have been introduced to control the introduction of new viruses. In the past, the Plum pox virus spread across Europe and North America in 1990, and then an extensive mitigation program was introduced to control the virus (Welliver, 2012). Two very recent examples are cassava mosaic disease and groundnut rosette disease in peanut, occurring after cassava and groundnut were introduced during the 16th century into Africa from South America (Carter et al., 1997; Naidu et al., 1999). A novel mastrevirus, sugarcane white streak virus (family *Geminiviridae*), was detected in quarantined sugarcane plants in France via NGS (Candresse et al., 2014).

A novel marafivirus and luteovirus were also detected in quarantined nectarine plants when they were probed using NGS-mediated detection (Bag et al., 2015; Villamor et al., 2016). The etiology of two separate citrus diseases has been updated through NGS. The first disease was found to be associated with a new alphaflexivirus, citrus yellow vein clearing virus, following siRNA analysis in Turkish accessions (Loconsole et al., 2012a). Moreover, siRNAs and complete DNA sequences were also analyzed to discover a highly divergent monopartite geminivirus, citrus chlorotic dwarf-associated virus (Loconsole et al., 2012b). Using NGS platforms, quarantine and certification programs have become more sophisticated, enabling the quick and disease-free dissemination of plant material across the globe (Figure 2).

## NGS and Characterization of Plant Viruses From Archeological Plant Material

The domestication of plants creates a new environment for the co-evolution of many pathogens. Most viral diseases have emerged recently and clues about their evolutionary pathway tracks have been lost in antiquity. The advent of NGS has enabled researchers to track the evolutionary footprints of such viral pathogens at the molecular level by sequencing ancient genomes from archeological materials. The re-construction of an archeological virus genome, that of barley stripe mosaic virus (BSMV), from a 750-year-old barley grain revealed that the divergence between the BSMV and its closest relative had taken place about 2000 years ago (Smith et al., 2014). Similarly, archeological material discovered in an approximately 50–168-year-old herbarium was found to contain peach latent mosaic viroid disease (Guy, 2013). Ancient maize cobs, dating from approximately 1000 CE from Antelope House in Arizona were found to contain the novel ds RNA plant virus genome of *Zea mays* chrysovirus 1 (ZMCV1), belonging to the plant and fungi infecting family, *Chrysoviridae* (Peyambari et al., 2019). These NGS analyses offer substantial new perspectives and knowledge on the evolutionary history of plant viruses and have helped to establish several new genera of plant viruses, such as the genera *Bymovirus*, *Macluravirus*, *Ipomovirus*, *Rymovirus*, and *Tritimovirus* (Gibbs et al., 2008).

## CRISPR-Cas-Mediated Resistance to Plant DNA Viruses

**FIGURE 3:**
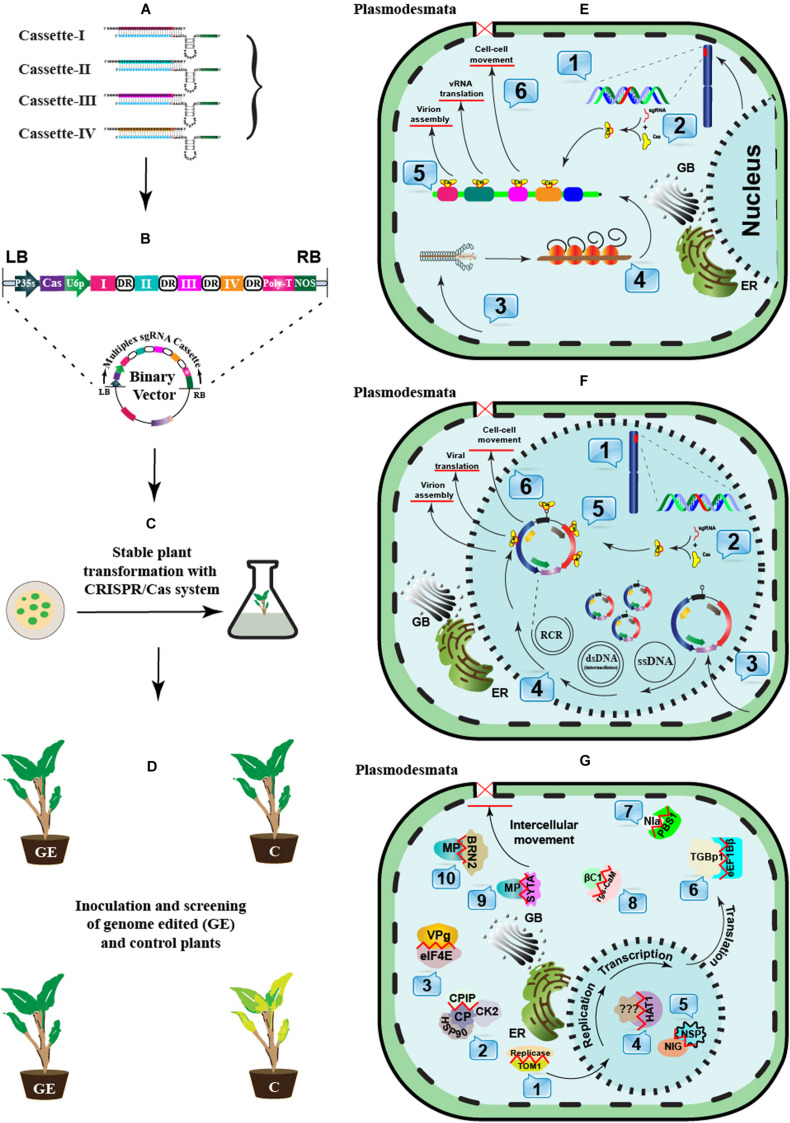
A schematic approach to engineer CRISPR-Cas-based antiviral resistance in crop plants. **(A)** Designing of sgRNA to target the virus-encoded genes or the host susceptibility factors. **(B)** Construction of a cassette to express multiple sgRNAs and Cas protein under suitable promoters. The Cas protein can be expressed under CaMV-35S promoter and NOS terminator sequences, while sgRNAs can be expressed under the RNA polymerase-III promoter. Direct repeat (DR) sequences can follow the cloning of individual sgRNAss. **(C)** A suitable recombinant binary vector carrying the multiplexed sgRNAs cassette can be employed for the stable *in vitro* genetic transformations in plants. **(D)** The successful GE plants are tested for resistance against single or multiple plant viruses through mechanical, *Agrobacterium*- or vector-mediated plant inoculation techniques. The plants expressing effective CRISPR-Cas machinery would show resistance against the invading viruses. **(E)** *In planta* CRISPR-Cas-based genetic resistance model against potato virus Y (PVY): 1. CRISPR-Cas components are transcribed in the successfully transformed plants. 2. The Cas protein and respective sgRNAs are assembled to make a complex. 3. Mechanical or vector-mediated transmission of PVY in the primary plant cell. 4. Uncoating and subsequent host-mediated translation of the viral RNA genome. Multiple viral proteins help in viral replication at the periphery of the endoplasmic reticulum. 5. The Cas:sgRNA complex recruits and subsequently binds to the targeted PVY genome. 6. The activation of the Cas:sgRNA complex disrupts the genes involved either in viral translation, virion assembly, or long-distance movement through plasmodesmata. **(F)** *In planta* CRISPR-Cas-based genetic resistance model against an ssDNA plant virus, cotton leaf curl Kokhran virus (CLCuKoV), and it associated DNA-satellites. 1. CRISPR-Cas components are transcribed in the successfully transformed plants. 2. The Cas protein and respective sgRNAs are assembled to form a complex. 3. *Agrobacterium*- or vector-mediated transmission of CLCuKoV and associated DNA-satellites in the primary plant cell. 4. The ssDNA viral genome replication in the cellular nuclei following rolling circle replication (RCR) via dsRNA intermediates. The Replication associated protein (Rep) initiates RCR and generates free 3’-hydroxyl end to prime ssDNA synthesis by nicking dsDNA molecules. 5. The Cas:sgRNAs complex recruits and subsequently binds to the targeted genes encoded by CLCuKoV and/or DNA-satellites genomes, respectively. 6. The activation of the Cas:sgRNA complex cleaves the viral genes involved either in viral translation, virion assembly, or long-distance movement through plasmodesmata. **(G)** A recessive resistance model mediated by host factors against plant viruses (RNA or DNA). During the infection cycle, plant viruses interact with several host factors to endure their successful infection. Many host factors are known as promising candidates in antiviral breeding, which do not perturb the plant development or growth if mutated. These host factors aid virus replication (1–3), transcription (4–5), translation (6–8), or intercellular long-distance movement (9–10). These host factors can be targeted through CRISPR-Cas based GE to break their interaction with viral proteins.

After successful detection, the development of broad-spectrum resistance can potentially limit infection by prevalent virus species and their variants. CRISPR-mediated viral immunity can be conferred directly by explicitly designing gRNA against the target virus(es) or indirectly by editing the host-susceptibility or resistance genes (Figure 3).

CRISPR-mediated resistance has successfully been executed against single-stranded (ss) DNA geminiviruses that replicate through dsDNA intermediates in the nuclei of host cells. The CRISPR-Cas system was first employed against two different mastreviruses, bean yellow dwarf virus (BYDV) (Baltes et al., 2015) and beet severe curly top virus (BSCTV) (Ji et al., 2015) in *Nicotiana benthamiana* and *Arabidopsis* plants, respectively. Both studies showed an up to 87% reduction in virus accumulation. A virus-inducible CRISPR-Cas system was later developed that transiently inhibited the BSCTV accumulation in *N. benthamiana* plants and the transgenic *Arabidopsis* plants with no off-target activity (Ji et al., 2018). In another study, tobacco rattle virus (TRV)-based vectors were used to express the CRISPR-Cas system into *N. benthamiana* plants to confer resistance against TYLCV by targeting three different regions of the TYLCV genome, including Rep protein, CP, and a non-coding intergenic region (IR). The construct targeting the IR region produced better results and more strongly inhibited virus replication than the other two constructs (Ali et al., 2015a). A similar approach targeting the IR region of cotton leaf curl disease-associated begomoviruses (CABs) has been proposed (Iqbal et al., 2016). A broad-spectrum resistance was achieved by targeting the IR region of three distinct begomoviruses, including cotton leaf curl Kokhran virus (CLCuKoV), TYLCV, and merremia mosaic virus (MeMV) (Ali et al., 2016). In another study, the simultaneous targeting of CLCuKoV-encoded Rep and βC1 of cotton leaf curl Multan betasatellite (CLCuMuB) via CRISPR-Cas9 system led to symptom attenuation and manyfold reduction in the viral titer (Khan et al., 2020). The simultaneous targeting of two regions, Rep and IR, of cotton leaf curl Multan virus (CLCuMuV) similarly produced almost complete resistance to CLCuMuV in *N. benthamiana* plants (Yin et al., 2019). Recently, a new Cas protein, Cas14a, to target ssDNA has been described (Khan et al., 2019); although no practical applications of this protein have been demonstrated in plants, it could potentially confer comprehensive resistance to geminiviruses and nanoviruses. Nonetheless, CRISPR-mediated resistance is not always successful. The AC2 and AC3 genes of African cassava mosaic virus (ACMV) were targeted but this did not result in resistance. The authors claimed that ACMV variants emerged after NHEJ repair and those variants triggered the rapid evolution of the virus (Mehta et al., 2019). The application of CRISPR-Cas9 has been extended to the dsDNA genome of cauliflower mosaic virus (CaMV) and a significantly lower viral titer was achieved in *Arabidopsis* plants by multiplexed targeting of the CP region (Liu et al., 2018).

## CRISPR-Cas-Mediated Resistance to Plant RNA Viruses

RNA viruses are the most diverse entity in the biosphere, and plant RNA viruses cause enormous economic losses to crop productivity worldwide. The advent of FnCas9, Cas13, and CasRx paved the way to restrict RNA virus infection in plants. The first-ever study to use two RNA viruses, TMV and CMV, expressed FnCas9 in *N. benthamiana* and *Arabidopsis* plants. Multiplexed GE was commenced by designing multiple gRNAs to target multiple regions of both viruses simultaneously. The results showed a 40–80% reduction in viral accumulation (Zhang et al., 2018). A theoretical approach to confer broad-spectrum resistance against PVY has been proposed recently (Hameed et al., 2019). Cas13 has successfully conferred immunity against plant-infecting RNA viruses. The first example of Cas13 utility was reported in 2018 against TuMV by transiently and stably expressing Cas13a in *N. benthamiana* plants (Aman et al., 2018a) but promising results could not be achieved. Later, the same researchers employed different Cas13 versions, including Cas13a, Cas13b, and CasRx (Cas13d), and the results revealed the robustness of CasRx against RNA viruses (Mahas et al., 2019a). CRISPR-Cas13 system was engineered in *N. benthamiana* plants to harness resistance against TMV and a significant attenuation in infection was associated with reduced viral titer (Zhang et al., 2020). Cas13 has also been successfully employed to engineer resistance in rice plants against two members of the negative-sense ss RNA rhabdoviruses, southern rice black-streaked dwarf virus (SRBSDV; genus *Fijivirus*) and rice stripe mosaic viruses (genus *Cytorhabdovirus*) (Zhang et al., 2019). In addition, Cas13-mediated resistance against a positive-sense ssRNA virus, potato virus Y (PVY, genus *Potyvirus*, family *Potyviridae*) has been achieved (Zhan et al., 2019).

## CRISPR-Cas-Mediated Host Genome Editing to Engineer Viral Resistance

CRISPR-Cas-mediated viral resistance can also be achieved by either mutating/inactivating the host susceptibility factors (S-genes) or expressing/activating resistance factors (R-genes). This approach involves commencing CRISPR-Cas-mediated modifications in the S- or R-genes. The transgenic sequences are then segregated from the progeny to yield non-transgenic plants. R-genes are usually linked to undesired traits such as poor flavor, low yield, or developmental abnormalities, meaning that they have the least potential in virus resistance. S-genes such as the *Arabidopsis ssi2* can confer resistance to CMV but exhibit similar problems including growth-related abnormalities. Despite this, most S-genes have promising potential for antiviral engineering. Inactivation of these susceptibility factors could lead to resistance without compromising plant general health due to the functional redundancy of the isoforms. Cao et al. (2020) categorized such S-genes into four groups (Cao et al., 2020). The first group comprised of negative regulators of plant defense like rgs-CaM (Li et al., 2018) and homeodomain leucine zipper protein 1 (HAT1) (Zou et al., 2016). The second group constitutes S-genes involved in different stages (such as replication/translation/movement) of the viral life cycle, for example, tobamovirus multiplication 1 (TOM1) and its homologs (Yamanaka et al., 2000), eEF1A and eEF4s, and Sec24a (a COPII coatomer) involved in replication, translation, and the movement of different viruses, respectively (Wang, 2015). Third group members interact and modify (phosphorylate) the viral proteins and include shaggy-related protein kinases (SK4-1, NsAK) (Lozano-Durán et al., 2011) and cellular CK2 (Lõhmus et al., 2017). The candidate host factors in the fourth group positively affect the virus behavior, such as secondary cell wall synthesis factor (Bearskin2B, BRN2) and phenylpropanoid metabolism factor (4-coumarate:CoA ligase1, 4CL1; Lozano-Durán et al., 2011).

Translation initiation factor *eIF4E*, and its isoforms, are among the most exploited susceptibility factors by the RNA viruses to induce a successful *in planta* infection (Sanfaçon, 2015). By opting for this technique, eif4 mutant plants were developed by transient expression of the CRISPR system and, subsequently, mutant plants demonstrated resistance to multiple potyviruses, including the zucchini mosaic virus, cucumber vein yellowing virus, and papaya ringspot virus (Chandrasekaran et al., 2016). The same *eIF4E* susceptibility factor of *Arabidopsis* was knocked out to confer resistance against TuMV (Pyott et al., 2016) and *eIF4G* was mutated in rice to curb rice tungro spherical virus (RTSV; family *Sequiviridae*) (Macovei et al., 2018). SK4-1 interacts with phosphorylate geminiviral C4 proteins to suppress disease symptoms (Lozano-Durán et al., 2011). Cassava-encoded *eIF4E* genes, nCBP 1 and 2, interact with the VPG protein of the cassava brown streak virus, and these two genes were mutated via the CRISPR-Cas system in cassava plants. The edited plants showed delayed and attenuated symptoms (Gomez et al., 2019). HAT1 nutation led to a higher accumulation of salicylic acid and jasmonic acid and resulted in resistance to CMV (Zou et al., 2016).

## Role of Different Cas Variants in GE Against Plant Viruses

**FIGURE 4:**
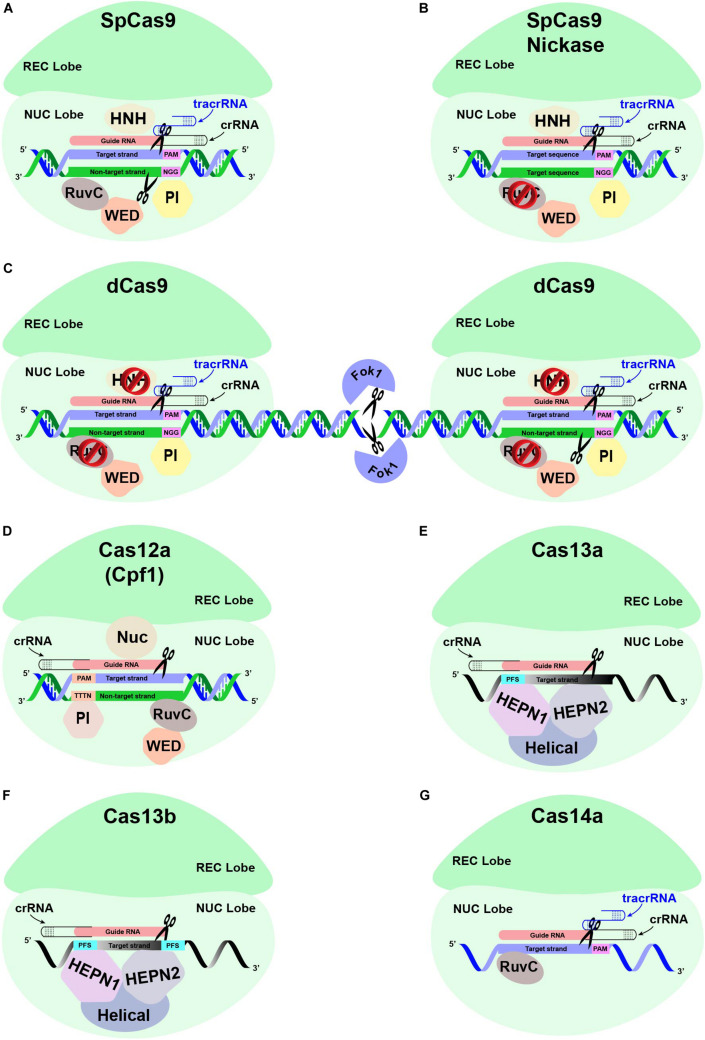
Different Cas proteins that have been opted widely to engineer antiviral resistance in plants. **(A)** The conventional CRISPR-Cas9-based genome editing is mediated through a single effector protein Cas9, the crRNA, and a tracrRNA. The crRNA and tracrRNA associated with Cas9 endonuclease are hybridized and subsequently bind to the target region with the help of ∼20 nucleotide (nt) guide RNA (gRNA) sequence upstream of the PAM sequence, respectively. The recognition lobe (REC) is responsible for recognizing this crRNA:tracrRNA:target DNA complex. The PAM interacting domain (PI) recognizes the PAM sequence. The HNH and RuvC domains in the NUC lobe cleave the target strand and the non-target strand through a blunt-ended double-stranded break (DSB) at the upstream of the PAM sequence, respectively. **(B)** The conventional Cas9 has been modified to reduce off-target mutations. SpCas9 is generated by introducing point mutations in one of the two nuclease domains, RuvC, which produces single-stranded breaks (SSB) rather than DSB. **(C)** The specificity of Cas9 protein is also enhanced by fusing catalytically inactive “dead-Cas9” (dCas9) to an RNA-guided Fok1 nuclease. **(D)** Unlike CRISPR-Cas9, the CRISPR-Cas12a-based GE is mediated through a single effector protein (Cas12a) associated with a single crRNA. The REC lobe recognizes the Cas12a:crRNA complex, which subsequently binds to the target strand specifically at the target region downstream of the PAM sequence. The PI domain recognizes the PAM sequence of ∼23–25 nt long gRNA, thus helping specific DNA-binding. A DSB is introduced at the target region, with the Nuc domain responsible for cleaving the target strand 18 nt downstream of the PAM, while the non-target strand is cleaved by RuvC domain 23 nt downstream of the PAM, respectively. **(E)** The CRISPR-Cas13-based GE is mediated via a single effector protein (Cas13) associated with single crRNA. The REC lobe is responsible for recognizing the Cas13 and crRNA complex, which binds to the recognition site based on sequence complementarity with the ssRNA substrate directed by the gRNA sequence of crRNA. The sequence-specific cleavage of the ssRNA substrate is mediated through HEPN1 and HEPN2 domains. Instead of PAM sequences, Cas13a protein is directed toward the ssRNA target via a single protospacer flanking sequence (PFS). **(F)** The CRISPR-Cas13b is distinct from Cas13a due to the presence of a suppressor and enhancer Cas genes directed by two PFS. **(G)** The CRISPR-Cas14a-based ssDNA GE is mediated via single-effector Cas14a in association with crRNA and a 130-bp-long tracrRNA. The crRNA:tracrRNA complex does not require the presence of the PAM sequence in the ssDNA substrates. Nevertheless, ssDNA cleavage by the RuvC domain requires sequence-specific complementarity of 20 nt in the crRNA guide sequence to the ssDNA substrate.

Several versions of CRISPR-Cas systems have been employed to engineer the genomes of different plant species (Table 2) after executing either DNA (SpCas9, SaCas9, Cas12a, and Csm1) or RNA (C2c2/Cas13a) targeting endonucleases (Figure 4). Some recently discovered endonucleases for DNA (Cas14a, Cas12e, and C2c1) (Harrington et al., 2018) or RNA (Cas13b) genomes could also be good candidates to study GE in plants. As most of the destructive plant viruses have ssDNA or ssRNA genomes, Cas14a and Cas13b can be of prime interest due to their ability to target ssDNA and ssRNA genomes, respectively. The average size of most of the endonucleases ranges between 400 and 1368 amino acids. Nonetheless, employing the smaller sized endonucleases such as SaCas9, Csm1, and Cas12e may improve the efficiency of the delivery of CRISPR-Cas components to the plant genome. Moreover, these miniature endonucleases can be stand-alone CRISPR effectors for virus-mediated GE against plant viruses (details in the preceding section). Additionally, Cas12a, Csm1, C2c1, and Cas12e endonucleases are specific for dsDNA genomes and thus produce staggered ends, making them useful for the homology-dependent repair (HDR) pathway.

**TABLE 2:** Examples of CRISPR-Cas-mediated genome editing into different plant hosts against different plant infecting viruses.

CRISPR-Cas system	Targeted virus(s)*	Genome	Genus	Family	Experimental host	References
CRISPR-Cas9	BSCTV	ssDNA	Curtovirus	*Geminiviridae*	*A. thaliana and N. benthamiana*	Ji et al., 2015
	CaLCuV	ssDNA	Begomovirus	*Geminiviridae*	Cabbage	Yin et al., 2015
	BSCTV	ssDNA	Curtovirus	*Geminiviridae*	*A. thaliana* and *N. benthamiana*	Ji et al., 2015
	BeYDV	ssDNA	Mastrevirus	*Geminiviridae*	*N. benthamiana*	Baltes et al., 2015
	TYLCV	ssDNA	Begomovirus	*Geminiviridae*	*N. benthamiana*	Ali et al., 2015a
	CVYV, ZYMV and PRSV-W	ssRNA (+)	Ipomovirus, Potyvirus	*Potyviridae*	*Cucumis sativus*	Chandrasekaran et al., 2016
	TuMV	ssRNA (+)	Potyvirus	*Potyviridae*	A. thaliana	Pyott et al., 2016
	TYLCV, MMV, BCTSV and BYDV	ssDNA	Begomovirus, Curtovirus, Mastrevirus	*Geminiviridae*	*A. thaliana* and *N. benthamiana*	Hadidi et al., 2016
	TRV and PEBV	ssRNA (+)	Tobravirus	*Virgaviridae*	*A. thaliana* and *N. benthamiana*	Ali et al., 2018
	TYLCV	ssDNA	Begomovirus	*Geminiviridae*	Tomato	Ghorbani et al., 2020
	Middle East-Asia Minor I (MEAM1)				*B. tabaci*	Heu et al., 2020
	ACMV	ssDNA	Begomovirus	*Geminiviridae*	*N. benthamiana*	Mehta et al., 2019
	CMV and TMV	ssRNA (+)	Cucumovirus Tobamovirus	*Bromoviridae, Virgaviridae*	*A. thaliana* and *N. benthamiana*	Zhang et al., 2018
	CaMV	dsDNA	Caulimovirus	*Caulimoviridae*	*A. thaliana*	Liu et al., 2018
	RTSV	ssRNA (+)	Waikavirus	*Sequiviridae*	*Oryza sativa*	Macovei et al., 2018
	TYLCV	ssDNA	Begomovirus	*Geminiviridae*	*N. benthamiana* and tomato	Tashkandi et al., 2018
	SMV	ssRNA (+)	Potyvirus	*Potyviridae*	Soya bean	Zhang et al., 2019
	TRV	ssRNA (+)	Tobravirus	*Virgaviridae*	*N. benthamiana*	Mahas et al., 2019a
	WDV	ssDNA	Mastrevirus	*Geminiviridae*	Barley	Kis et al., 2019
	ChiLCV	ssDNA	Begomovirus	*Geminiviridae*	*N. benthamiana*	Roy et al., 2019
CRISPR-Cas13/a	PVY	ssRNA (+)	Potyvirus	*Potyviridae*	Potato	Zhan et al., 2019
	TuMV	ssRNA (+)	Potyvirus	*Potyviridae*	*A. thaliana*	Aman et al., 2018b
	TuMV	ssRNA (+)	Potyvirus	*Potyviridae*	*N. benthamiana*	Aman et al., 2018a
	PVY	ssRNA (+)	Potyvirus	*Potyviridae*		Hameed et al., 2019
	RNA viruses	ssRNA (+)	Potyvirus	*Potyviridae*	*N. benthamiana*	Chaudhary, 2018
	Plant pathogens/pest				Soybeans	Abudayyeh et al., 2019
CRISPR-Cas14/a	Geminiviruses	ssDNA	Begomovirus	*Geminiviridae*		Khan et al., 2019
	Geminiviruses	ssDNA	Begomovirus	*Geminiviridae*		Aquino-Jarquin, 2019
	Geminiviruses	ssDNA	Begomovirus	*Geminiviridae*		Harrington et al., 2018
CRISPR-CasRx	RNA viruses	ssRNA (+)	Potyvirus	*Potyviridae*	*N. benthamiana*	Mahas et al., 2019a

*Viruses acronyms used are: African cassava mosaic virus (ACMV), bean yellow dwarf virus (BeYDV), beet curly top virus (BCTV), beet severe curly top virus (BSCTV), cabbage leaf curl virus (CaLCuV), chilli leaf curl virus (ChiLCV), cauliflower mosaic virus (CaMV), cucumber vein yellowing virus (CVYV), cucumber mosaic virus (CMV), merremia mosaic virus (MMV), papaya ring spot mosaic virus-W (PRSV-W), pea early browning virus (PEBV), potato virus Y (PVY), rice tungro spherical virus (RTSV), soybean mosaic virus (SMV), tobacco mosaic virus (TMV), tobacco rattle virus (TRV), tomato yellow leaf curl virus (TYLCV), turnip mosaic virus (TuMV), wheat dwarf virus (WDV), and zucchini yellow mosaic virus (ZYMV). Virus genomes are denoted as single-stranded (ss) DNA and a positive sense (+) ssRNA.

### Cas9 Nuclease

Cas9 (earlier known as Cas5, Csn1, or Csx12) plays a significant role in the defense of certain bacteria against viruses and plasmids. Cas9 is an RNA-guided DNA endonuclease enzyme associated with the CRISPR adaptive immune system in bacteria. Despite its original function as a defense protein, Cas9 has been greatly exploited as a genome engineering tool to induce site-directed double-strand breaks in DNA. Cas9 induces dsDNA breaks in a sequence-specific manner and is directed to a target locus by a gRNA (Jinek et al., 2012; Gilbert et al., 2013; Maeder et al., 2013). The CRISPR-based GE cleavage complex requires tracrRNA besides Cas9 and crRNA, where crRNA and tracrRNA are mutually expressed as a sgRNA molecule (Jinek et al., 2012). CRISPR-Cas9 could be reprogrammed for another target site within the genome by changing the sequence of its crRNA.

### Cas12a Nuclease

The Cas12a (Cpf1) nuclease belongs to the class-II type V CRISPR system. Contrary to the Cas9 nuclease, which requires two separate catalytic domains, *viz*., HNH and RuvC, Cas12a only need one catalytic domain (RuvC) for creating DSB in DNA. Cas12a only needs crRNA to be fully operational instead of crRNA and tracrRNA duo, which is a must for Cas9. The efficiency of Cas12a further improves as it produces sticky ends by generating 5′ overhangs during DSBs compared to the blunt end, a characteristic of the Cas9 system (Zetsche et al., 2015; Fonfara et al., 2016). The idea of the generation of 5’ overhangs is further strengthened by the fact that HDR mechanisms prefer sticky ends (Vu et al., 2017; Schmidt et al., 2019).

### Cas13 Nuclease

Cas13 proteins have recently been re-programmed to target ssRNA viral genomes infecting plants (Mahas et al., 2019b) and humans (Freije et al., 2019) without any known off-targets. The DNA-nuclease activity of Cas13 orthologs (Cas13a, Cas13b, and Cas13d) is replaced with two Higher Eukaryotes and Prokaryotes Nucleotide-binding (HEPN) domains. The HEPN domains have exclusively transformed Cas13 as an RNA-guided RNA endonuclease to edit ssRNA targets specifically. Cas13 is explicitly a single effector nuclease, which acts as a dual-effector nuclease mediating the processing and maturation of precursor-CRISPR (pre-CRISPR) RNA into CRISPR RNA (crRNA), respectively. Thus, a single crRNA of Cas13 proteins mediates the recognition of target ssRNAs following an RNA–RNA hybridization (Wessels et al., 2020). The two HEPN domains mediate the nuclease function of Cas13a, preferably at U-rich targets. Empirically, a single point mutation at any HEPN domain can abolish the nuclease activity of Cas13a, resulting in a catalytically inactive dead Cas13a (dCas13a). Interestingly, dCas13a can retain the RNA-binding ability with higher sensitivity. Contrarily, the sequence-specific RNA cleavage activity of Cas13b depends upon double-sided protospacer flanking sequence (PFS) sites and ssRNA templates (Hameed et al., 2019). Cas13 nucleases may commit some non-specific nuclease activity; however, their off-target mutations are greatly reduced compared to RNAi (Abudayyeh et al., 2017). Although the PFS constraints are minimal in the Cas13 gRNAs design, the spacer sequences are mostly intolerant for any mismatches between 15 and 21 nt (Wessels et al., 2020). Thus, the applications of catalytically active Cas13a have been extended into the plant genomes and targeting ssRNA viruses such as potyviruses (family *Potyviridae*). These studies showed that Cas13a conferred target-specific resistance against TuMV in *Arabidopsis* and *N. benthamiana* plants (Aman et al., 2018a; Mahas et al., 2019a). Both Cas13a and Cas13b showed robust and highly specific interference against multiple RNA viruses (Mahas et al., 2019a), whereas Cas13d conferred the most robust interference against RNA viruses in transient assays as well as *in planta*, respectively (Mahas et al., 2019b). Moreover, Cas13d has been shown to target two RNA viruses simultaneously and could be a good choice in enhancing broad-spectrum resistance against single and multiple viruses in plants.

### Cas14 Nuclease

It was recently discovered that Cas14 nucleases are effector proteins in extremophile archaea, which cleave only ssDNA instead of dsDNA or ssRNA (Harrington et al., 2018). These class 2, type V nucleases are notably smaller in size (400–700 amino acids) compared to other class 2 counterparts. Unlike other class 2 Cas effectors, PAM binding is not an absolute requirement for Cas14 nucleases for their cleavage activity (Savage, 2019). Nevertheless, the gRNA must have a 20 bp sequence complementary to the target ssDNA. Cas14 nucleases require certain sequence specificity in the central 6 bp stretch of the gRNA to be activated. Moreover, RNase-III endonuclease activity is missing in the Cas14 system (Harrington et al., 2018). The Cas14-mediated ssDNA cleavage can help to confer resistance against viruses with an ssDNA genome or mobile genetic elements (MGEs) such as viruses, plasmids, and transposons (Aquino-Jarquin, 2019). These characteristics make Cas14 nucleases uniquely suitable for nucleic acid detection. More specifically, Cas14 is capable of recognizing SNPs with high-fidelity in the targeted region.

## Role of NGS in the Screening of GE Events and the Evaluation of GE Efficiency

Next-generation sequencing and CRISPR-Cas systems can be executed one after the other while conferring resistance to plant viruses. After successfully editing the susceptibility factor, eIF4E4, of the *Arabidopsis* genome, Pyott et al. (2016) performed extensive screening to yield transgene-free plants followed by developing homozygous mutations into T2 and T3 generations. Similarly, Macovei et al. (2018) carried out extensive screening to produce the transgene-free GE rice plants.

Although CRISPR-mediated plant genome engineering shares unparalleled advantages, it still poses two major pitfalls that need to be addressed in every successful event and are a major constraint in commercializing GE crops. The first challenge is the presence of a transgene, the second is unwanted (off-targets) mutations, and both pose a high risk to biosecurity. In plants with a shorter life span, it is easy to remove most of the CRISPR-Cas cargo via backcrossing and/or screening of segregating populations, but this approach is impractical for vegetatively propagated and longer life-spans plants. Additionally, achieving transgene-free plants is trickier as it may take years to get transgene-free seeds. One effective way to reduce the transgene integration is to express CRISPR-Cas system transiently. However, complete elimination is highly unlikely and the resulting degraded DNA fragments can still be integrated into the plant genome (Zhang et al., 2016). Therefore, developing transgene-free plants without any off-targets is significant, as this will persuade common customers and policymakers that the genome of GE plants is comparable to the native plants. To address these notions, NGS offers CRISPR-induced mutations (both on- and off-target), with a quick selection of transgene-free GE plants. Thus, it is unbiased, direct, and potentially can identify SNPs, indels, and different kinds of structural variants such as genome shuffling, inversions, and duplication (Manghwar et al., 2020).

The NGS platform has been exploited to detect on- and off-target changes in a variety of plant species including rice (Zhang et al., 2014; Tang et al., 2018), *Arabidopsis* (Feng et al., 2014; Xu et al., 2019), cotton (Li et al., 2019b), and tomato (Nekrasov et al., 2017). An assay compared the editing efficiency of T7E1, Indel Detection by Amplicon Analysis (IDAA), Tracking of Indels by Decomposition (TIDE), and targeted NGS in mammalian cells, where T7E1 often does not produce precise results but NGS does. TIDE and IDAA can also skip the alleles in edited clones compared to NGS (Sentmanat et al., 2018). Based on Illumina sequencing followed by a high-resolution melting (HRM) analysis, a cost-effective, rapid, and high-throughput mutant screening protocol was developed in tetraploid tobacco plants. In this study, CRISPR-mediated GE of *Phytoene desaturase* (PDS) gene was achieved, followed by regeneration and sequencing. The results of one study revealed that 17.2% of plants were non-transgenic, so the established method was unprecedented in the development of non-transgenic GE plants without segregating out the transgenes via sexual reproduction (Chen et al., 2018).

To improve the role of host genetic resources to confer resistance against plant viruses, the screening of host factors is essential. However, traditional approaches such as random mutagenesis and introgression through crossings are labor-intensive, costly, and difficult to execute due to functional redundancy. Moreover, these host factors have an essential role in plant viability and can lead to lethal phenotypes (Nicaise et al., 2007; Patrick et al., 2014). Nevertheless, NGS and CRISPR-Cas have opened new avenues for identifying, improving, and executing genetic resources for host-factors-mediated viral resistance. Such loci have been identified via NGS from *Arabidopsis* and *Gossypium hirsutum* (Schneeberger et al., 2009; Austin et al., 2011; Hashimoto et al., 2016). Most of the S-genes (discussed in section “CRISPR-Cas Mediated Host Genome Editing to Engineer Viral Resistance” and Figure 3G) are excellent candidates for antiviral engineering due to their functional redundancy and isoforms.

## Applications of CRISPR-Cas-Based Biosensing Technology in Plant Virology

The CRISPR-Cas-based system can be harnessed to develop biosensing systems. Cas proteins can be fused with a split enzyme or a split fluorescent protein to build a biosensing system. Various CRISPR orthologs have been optimized to develop a cost-effective, highly sensitive, and pathogen-specific diagnosis of infectious and non-infectious diseases (Li L. et al., 2019). These biosensing platforms are DNA- or RNA-based CRISPR-Cas effectors. A remarkable collateral activity of Cas12a is the complete cleavage of ssDNA molecules during its binding to the target dsDNA. This non-specific cleavage activity is the basic principle of DNA endonuclease targeted CRISPR trans-reporter (DETECTR) development, where Cas protein (Cas12a or Cas14) is combined with the isothermal amplification technique such as recombination polymerase amplification to detect target DNA that subsequently cleaves ssDNA sequences coupled to fluorescent reports. DETECTR has been successfully employed in clinical diagnostics (Ai et al., 2019). Other applications of Cas12 have led to the development of 1-h low-cost multipurpose highly efficient system (HOLMES) and its derivative HOLMESv2 for rapid detection of target DNA/RNA and SNPs, respectively (Li L. et al., 2019). HOLMESv2 has been further improved for accurate quantification of DNA methylation in combination with Cas12b nuclease. The high-throughput application of Cas12 nuclease has been further expanded to develop CaT-SMelor (CRISPR-Cas12a- and a transcription factor-mediated small molecule detector) to detect small molecules and to discriminate them from their structural analogs (Liang et al., 2015). Furthermore, Cas14 has expanded promising high-fidelity SNPs genotyping tools to detect the ssDNA viruses infecting eukaryotes (Aquino-Jarquin, 2019). More interestingly, the ability of Cas14 to detect ssDNA targets independent of PAM recognition makes it an excellent candidate for detecting ss- and dsDNA. The non-specific DNase activity of Cas14 has been utilized to develop the Cas14-DETECTR method, which is more specific and active than its counterpart (Cas12a-DETECTR) as a high-fidelity system for DNA SNPs (Harrington et al., 2018). Cas14-DETECTR can be exploited for high-fidelity SNPs genotyping and detection of ssDNA viruses. Finding low abundance sequences by hybridization (FLASH) is another next-generation diagnostic tool based upon the specificity of Cas9 to efficiently enrich specific target sequences (Quan et al., 2019).

The discovery of Cas13 and its derivative nuclease has led to a variety of RNA-based applications in different systems due to its dual enzymatic activity (i.e., pre-crRNA processing and signature HEPN domain). These include various biosensing applications such as the detection of viral RNA (Gootenberg et al., 2017; Mahas et al., 2019b), precise RNA editing (Cox et al., 2017), site-directed mRNA demethylation (Li et al., 2019a), dynamic real-time RNA imaging (Wang et al., 2019), and site-specific polyadenylation in eukaryotic cells (Anderson et al., 2019). During the binding and cleavage of target RNA, the activation of Cas13 triggers random collateral trans-ssRNA cleavage in its vicinity. East-Seletsky et al. (2016) used the collateral ssRNA cleavage activity of Cas13 to detect the presence of specific nucleic acids by constructing reporter RNAs and the release of fluorescent signals upon recognition and cleavage (East-Seletsky et al., 2016). Further refinement in this technique led to the SHERLOCK development (specific high-sensitivity enzymatic reporter unlocking) detection method (Gootenberg et al., 2017; Kellner et al., 2019). The SHERLOCK method has been successfully utilized to detect specific strains of Dengue and Zika viruses. A more refined version of SHERLOCK is heating unextracted diagnostic samples to obliterate nucleases (HUDSON) which allows the detection of a low titer of a pathogen in a biological sample (Myhrvold et al., 2018). Further improvement in the SHERLOCK system resulted in SHERLOCKv2, which combined different Cas proteins (such as Cas12 and Cas13) in a single reaction, enabling multiplexed nucleic acid detection. The sensitivity of SHERLOCKv2 was further improved by joining Csm6 nuclease to amplify the signal of Cas13 collateral cleavage and the development of a FAM-biotin reporter kit (Gootenberg et al., 2018). The application of SHERLOCKv2 has been further expanded for various agricultural applications such as genotyping and quantification of genes related to pathogen resistance (Abudayyeh et al., 2019). The SHERLOCKv2 can be optimized exponentially for the detection of vital traits, surveillance of insect pests and disease, or other agricultural applications (Aman et al., 2020). The amenability of CRISPR-Cas-based multiplexing systems can enable biosensing techniques to identify multiple target nucleic acids (even at low concentrations) through a single diagnostic test kit. Further refinements may lead to cost-effective, super-sensitive, highly accurate, and on-field diagnostic kits with a range of applications in agriculture.

## Plant Viruses as CRISPR Delivery Vectors

The targeting efficiency of CRISPR-Cas depends on the efficient delivery of the CRISPR-cassette. This is usually achieved via biolistic or *Agrobacterium*-mediated plant transformation. In either case, only a limited number of cells are transformed and extra cargo like promoters and the presence of degrading DNA molecules give rise to GMO concerns. To this end, more efficient delivery methods are required to integrate the GE reagents effectively into economically important crops. The use of virus-based vectors with autonomous replication is popular for integrating GE cassettes into target plants with improved efficiency (Zaidi and Mansoor, 2017). Most importantly, these viral vectors with either a DNA or RNA genome have been effectively employed to harness GE in model plants and commercial crops. Plant viruses are an attractive option for gene delivery to the host genome due to their intrinsic proximity with the host cells, their autonomous mode of replication, and smaller structure (Pasin et al., 2019).

Plant viruses are being used as vectors to express foreign proteins and RNAs (Cody and Scholthof, 2019). SSRNA viruses such as TRV and TMV, and ssDNA viruses of the family *Geminiviridae* such as BYDV, cabbage leaf curl virus, and wheat dwarf virus (WDV) have been employed for efficient GE. Geminiviruses have characteristics such as a wide host range, a single replication associated protein (Rep) for *in planta* replication, and the production of many replicons inside host cells (Hanley-Bowdoin et al., 2013). To overcome their limited cargo capacity, the geminiviruses were engineered as non-infectious but replicating systems. The movement protein (MP) and coat protein (CP) were substituted with the Cas protein and the sgRNA sequences (Čermák et al., 2015) to achieve hyper-expression of the CRISPR-Cas system; however, the absence of CP and MP limits their application for only transient expression at localized tissues. The MP can be expressed transiently using another vector or a bipartite genome of a begomovirus (which encodes MP on a separate genomic component) to overcome this problem. Another possibility is to engineer geminivirus associated DNA-satellites to deliver the GE components. These DNA-satellites have been successfully modified as virus-induced gene silencing (VIGS) vectors for several crops and can be a good choice as cargo vectors for GE reagents.

Tobacco rattle virus-based vectors are simple, versatile, and efficient GE tools that surpass long transformation and tissue culture procedures. Such vectors are equally effective for cloning, multiplexing, library construction, and agroinfiltration. The most important feature of TRV-based vectors is the possibility of producing transgene-free GE plants because their RNA genome is not integrated into the plant genome (Mahas et al., 2019a). However, the TRV-based CRISPR-Cas systems are limited to the transgenic lines of those crops in which Cas9 protein is stably expressed. TMV-based ssRNA vectors have also been used for the transient expression of certain genes and offer flexible gene expression and GE in a variety of crops (Cody et al., 2017). Kaya et al. (2017) developed a modified version of a TMV-based expression vector to express the target region of tobacco microRNA 398 (miR398). After plant transformation, during shoot development in the regenerated plants, the miR398 expression eliminated the viral RNA. Moreover, a split-protein approach has been used to transiently express the Cas9 protein from two fragments via TMV and *Agrobacterium*. The active Cas9 protein was re-assembled and ultimately induced a targeted mutation producing virus-free GE plants via tissue culture (Kaya et al., 2017). The utility of viral vectors for GE has opened a new era of functional genomics and applications in agricultural biotechnology. However, disposing of the viral genome from the GE plants may pose a serious challenge at a later stage. The use of meristematic tissue culturing can be a possible solution to eliminate the virus and its remnants from GE plants.

## Bottlenecks in Adoption of NGS and GE Tools in Plant Virology

Despite these innovative applications, CRISPR-Cas-based GE still has some limitations in terms of translational research applications, especially in engineering resistance to plant viruses. The efficient delivery of recombinant plasmids into the host genome followed by the successful regeneration of plants is a challenging task, particularly in the vegetatively propagated plants. Stable plant transformation is the key to regenerating transgenic plants with GE events and heritable mutations. Plasmid transgenes are usually segregated out of the developing progenies of the transformed plants at a later stage to make them transgene-free. However, this strategy does not apply to all crop plants. Moreover, if the designed sgRNAs are based on the potential plant virus genome, these cannot be segregated out from the developing plant progenies. Alternatively, the application of DNA-free GE techniques can be used. The popular biolistic and *Agrobacterium*-mediated plant transformation is not competent for many crop plants in terms of (1) low frequency of transformation, (2) prolonged tissue culturing procedures, (3) impaired tissues during biolistic delivery, (4) limited application of *Agrobacterium*-mediated plant transformation, (5) tissue browning and necrosis due to *Agrobacterium*, (6) somaclonal variations in the regenerated plants, and (7) insufficient DNA-delivery to induce HDR. The optimization of certain explants and culturing media are required to bypass these problems (Altpeter et al., 2016).

A major setback of GE in plants is the primary mode of DNA-repair via NHEJ, which produces many unwanted genetic mutations compared to HDR. The most significant cellular DNA-repair pathway during GE is HDR, which requires high titers of nucleases and repair templates delivered into the targeted plant tissues or cells. Enhancement of the efficacy of HDR in plants during a GE event is required. Plant viral-mediated transformation strategies can be a good alternative to deliver nucleases and sgRNAs into the host plant genome for increased HDR frequency during GE. TRV-based plant transformation vectors have been developed recently for successful GE in many plant species (Ali et al., 2015b). TRV-based transformations are crucial because the RNA genome of TRV vectors could not be integrated into the plant genome and could lead to transgene-free plant transformations (Mahas et al., 2019a). The use of a marker-free transformation technique using regeneration promoting factors (cycD3, auxin, and cytokinin-related genes) can also be used to apply GE to a wide range of plant species (Vats et al., 2019).

## Critical Assessment of CRISPR-Cas and NGS Techniques in Plant Virology

Despite the popularity and tremendous success of the CRISPR-Cas technology, there are still some limitations to its application in crop plants. These include but are not limited to, off target effects, the difficulty of execution in woody plants, low mutagenesis, inefficient delivery approaches, dependence on *in vitro* regeneration, persistent activity in subsequent generations, potential risk of transgene transfer to wild type population, risk of reversion of induced mutations via cross-pollination, and stringent GMO regulations.

Most of the resistance strategies targeting the virus genome have failed to provide laconic control due to their inherent inability to target multiple and synergistically interacting viruses. Other reasons may include the evolution of the viral genomes through mutations and plant viruses, which form sub-genomic components to evade host resistance. To circumvent such problems, CRISPR-Cas technology holds great potential as an effective antiviral technology that can not only target the viral genome at multiple sites but can also simultaneously target different, related, or unrelated virus genomes. Aside from directly targeting the viral genome, the CRISPR-Cas can be executed to nullify the host susceptibility factors and circumvent the problem of generating resistance to viral mutants.

Despite these numerous successful applications, the execution of CRISPR-Cas still faces many challenges. It requires a meticulous and critical approach to avoid erroneous design. Various strategies have been proposed to address off target activity, such as GC contents should be ideally between 40 and 60% to form stable DNA:RNA duplex in the gRNA and to enhance the on-target activity and destabilize off-target binding (Wang et al., 2014). Higher GC contents (65–80%) can lead to off-target activity. The length of gRNA can have a profound effect on GE efficiency and can lead to off-target/unwanted mutations; for example, the results of studies appraising the 16-to-20 nucleotides long gRNA effect on GE efficiency and off-target mutations showed higher GE efficiency when 18–20 bp long sgRNAs were used (Fu et al., 2014; Sugano et al., 2018). Furthermore, the dead RNA off-target suppression (dOTS) technique employs dead truncated gRNA, which can guide Cas9 while suppressing cleavage, reducing off-target activity, and improving on-target activity by 40-fold (Rose et al., 2020). The chemical modification of gRNA, by incorporating 20-*O*-methyl-30-phosphonoacetate into the gRNA ribose-phosphate backbone, improves the on-target efficiency by 40–120-fold (Ryan et al., 2017).

Using a low concentration of Cas protein/gRNA is another potential way to minimize off-target effects. The expression of Cas9 under the control of CaMV35S (constitutive) promoter and an egg-cell (inducible) promoter (ECS) was evaluated. The results demonstrated that constitutive expression via CaMV35S promoter revealed a low editing frequency compared to ECS promoter (Begemann et al., 2017). Likewise, the use of embryo-specific promoters (YAO) yielded better and more efficient GE in *Citrus sinesis* (Zhang et al., 2017). The use of different Cas proteins variants has a substantial impact on the reduction of off-target effects. A comparative study of Cas9 and Cas12a revealed that the Cas9 mechanism is more specific, efficient, and accurate (90–100%) than Cpf1 (0–60%) in maize plants (Lee et al., 2019). Similarly, modified variants like dead or deactivated Cas variants (dCas) have fewer off target activities (Brocken et al., 2017).

The delivery of the CRISPR-Cas system is one of the vital factors for achieving better on-target and the least off target activity. Several transformation methods such as PEG-, *Agrobacterium*-, biolistic-, protoplast, ribonuclease protein (RNP) complex-, lipid- and polymer-, and viral vectors-mediated methods are being practiced. These approaches, however, are not free from drawbacks and share limitations. Another ailment to CRISPR-Cas utility is the persistence of Cas activity in subsequent generations that can induce unanticipated mutations in stable lines and has been reported in *Arabidopsis*, cotton, and maize plants (Feng et al., 2014; Char et al., 2017; Wang et al., 2018). To circumvent this problem, different strategies have been employed, such as the use of transgene-free viral vectors for cassette delivery and *in vitro* expression of pre-assembled gRNA:Cas (RNP) complex (Woo et al., 2015; Zhang et al., 2016). RNP complex can yield Cas protein-free GE cells because it is degraded quickly after cleaving the target site, meaning that it can address persistent concerns about Cas activity. However, degraded Cas protein and degraded DNA fragments can still induce some undesired mutations at a lower frequency. The Transgene Killer CRISPR (TKC) technique is another promising tool to yield transgene-free GE plants (He et al., 2018). The TKC technique uses the temporal expression of the Cas protein and suicide genes; the former is expressed first at the transformation (at callus formation and organogenesis) stage, while the latter is expressed at the embryogenesis stage to kill all the pollens and embryos with the transgene. Eventually, transgene-free plants are yielded without labor-intensive screening and selection. These technologies will minimize the regulatory GM burden, mitigate ecological challenges, and foster public acceptance of GE plants and related byproducts.

Base editors (BEs) are another substantial addition to the CRISPR toolbox that have enabled site-specific modification (base substitution) without inducing DSBs. BEs and prime editors (Pes) are moving to the front lines of precision genetic engineering. In base editing, all four transition mutations (C→T, G→A, A→G, and T→C) can be achieved. The Adenine BE can substitute A→G, and the Cytosine BE can substitute C→T at the target site. These BEs can be employed to confer antiviral resistance by introducing stop codons in the coding regions of viral genomes via iSTOP (Billon et al., 2017) or CRISPR-stop (Kuscu et al., 2017) technologies. The resulting viral proteins will be non-functional and limit viral spread. Similarly, base editing could be used to develop plants with immunity against different single and multiple pathogens by targeting and modifying the host susceptibility factors (S-genes).

High copy numbers in polyploidy plants pose unique challenges, such as knock-out of all copy numbers with equal efficiency and of genes with high homology. To achieve a plant with all copy number mutations, a series of allelic variants are first executed, and then a subsequent selection is performed in the segregating population (Vats et al., 2019). CRISPR-Cas-based GE has enabled easy editing and the introgression of multiple traits in polyploid plants without any linkage drag, which otherwise is a tedious and laborious task through conventional breeding approaches.

In the plant virus-combating arsenal, the interaction and battle between host plants and viruses resemble a never-ending arms race. Viral genomes are dynamic entities so that CRISPR-Cas-mediated resistance can facilitate and speed up the evolution and generation of new viral variants. Some studies have already reported such notions in ACMV (Mehta et al., 2019), CLCuKoV, MeMV, TYLCV (Ali et al., 2016), and CaMV (Liu et al., 2018). However, the combinations of two single gRNAs and/or targeting non-coding IR regions (in the case of geminiviruses) resulted in a substantial delay in resistance breakdown (Ali et al., 2016).

Next-generation sequencing, as a go-to tool for plant virologists, has shaped plant virology by sequencing whole virus genomes, undertaking plant metagenomics studies, and characterizing viruses from archeological and quarantined plant samples. Since GE requires pre- and post-knowledge of the target site, the former is needed to precisely carry out the GE, while the latter is required to evaluate both on- and off-target efficiency. In this context, NGS has been quite instrumental, but the availability of the whole genome is just limited to a few plant species (mostly model plants). The successful execution of CRISPR-Cas in plants requires knowledge of genetic variations, chromosomal rearrangements, indels, SNPs, transposon occurrence, and copy number variations. NGS and whole-genome sequences are thus a prerequisite not just in model systems, but for all plant species for which antiviral systems are engineered.

Next-generation sequencing can demonstrate the characterization of latent viruses or viruses that are of less concern regarding agricultural production. Nevertheless, if such viruses evolve, adapt, and become an emerging threat in the future, then NGS revelation will undoubtedly help to develop rapid diagnostic assays and better management strategies. One challenge, however, lies in the cost and processing of NGS data, which requires sophisticated machines, tools, and expert personnel.

## Concluding Remarks

Although NGS technologies have been evolving swiftly over the last decade, there are a variety of parallel options being practiced, particularly for virus characterization. Historically, ELISA in the 1980s and later PCR-based approaches in the 1990s contributed to detecting plant viruses and disease etiologies. However, NGS has enabled the detection and characterization of novel plant viruses that have remained undetectable by conventional diagnostic approaches. Recent versions of NGS technologies such as PacBio by Illumina, Oxford Nanopore, and ISS may significantly boost plant virology by providing faster, more reliable virus detection with reduced errors and direct RNA sequencing.

The CRISPR-Cas toolbox has a range of tools for GE and is still expanding so that almost all types of viral genomes can be targeted/engineered. In the field of plant virology, CRISPR-Cas provides a versatile platform that can be engineered for biosensing, detection of small molecules, site-directed mutagenesis, genotyping, SNPs detection, gene quantification, and substitution of a single nucleotide. After successfully executing CRISPR-Cas systems in plant species, extensive screening to yield transgene-free plants is required and NGS platforms have been used to detect on- and off-targets in several crops. Based on Illumina sequencing followed by an HRM analysis, a cost-effective and high-throughput mutant screening protocol can be developed for different crop plants, as a similar approach has been furnished in tetraploid tobacco plants where the PDS gene was targeted. NGS, coupled with CRISPR-Cas, has already contributed to the control of plant viral diseases. In the near future, basic biological issues for antiviral engineering will be addressed through CRISPR-Cas-based technologies and the current GMO-related concerns of the common people may be nullified.

## Author Contributions

MSh, AR, MSa, and ZI conceived the ideas and wrote and finalized the manuscript. MSh drew tables. MSa and ZI drew figures. AA-S prepared the final version. Finally, all authors proofread and approved the manuscript.

## Conflict of Interest

The authors declare that the research was conducted in the absence of any commercial or financial relationships that could be construed as a potential conflict of interest.
